# Mechanical Response of Porcine Liver Tissue under High Strain Rate Compression

**DOI:** 10.3390/bioengineering6020049

**Published:** 2019-05-30

**Authors:** Joseph Chen, Sourav S. Patnaik, R. K. Prabhu, Lauren B. Priddy, Jean-Luc Bouvard, Esteban Marin, Mark F. Horstemeyer, Jun Liao, Lakiesha N. Williams

**Affiliations:** 1 Department of Biological Engineering and Center for Advanced Vehicular Systems, Mississippi State University, Mississippi State, MS 39762, USA; chen.joseph@berkeley.edu (J.C.); sourav.patnaik@utsa.edu (S.S.P.); rprabhu@abe.msstate.edu (R.K.P.); lbpriddy@abe.msstate.edu (L.B.P.); jean-luc.bouvard@mines-paristech.fr (J.-L.B.); marineb@corning.com (E.M.); marineb@corning.com (M.F.H.); 2 Department of Bioengineering, University of Texas at Arlington, Arlington, TX 76010, USA; 3 Department of Biomedical Engineering, University of Florida, Gainesville, FL 32611, USA

**Keywords:** soft tissue, liver, high-rate compression, polymeric split-Hopkinson pressure bar, finite element modeling

## Abstract

In automobile accidents, abdominal injuries are often life-threatening yet not apparent at the time of initial injury. The liver is the most commonly injured abdominal organ from this type of trauma. In contrast to current safety tests involving crash dummies, a more detailed, efficient approach to predict the risk of human injuries is computational modelling and simulations. Further, the development of accurate computational human models requires knowledge of the mechanical properties of tissues in various stress states, especially in high-impact scenarios. In this study, a polymeric split-Hopkinson pressure bar (PSHPB) was utilized to apply various high strain rates to porcine liver tissue to investigate its material behavior during high strain rate compression. Liver tissues were subjected to high strain rate impacts at 350, 550, 1000, and 1550 s^−1^. Tissue directional dependency was also explored by PSHPB testing along three orthogonal directions of liver at a strain rate of 350 s^−1^. Histology of samples from each of the three directions was performed to examine the structural properties of porcine liver. Porcine liver tissue showed an inelastic and strain rate-sensitive response at high strain rates. The liver tissue was found lacking directional dependency, which could be explained by the isotropic microstructure observed after staining and imaging. Furthermore, finite element analysis (FEA) of the PSHPB tests revealed the stress profile inside liver tissue and served as a validation of PSHPB methodology. The present findings can assist in the development of more accurate computational models of liver tissue at high-rate impact conditions allowing for understanding of subfailure and failure mechanisms.

## 1. Introduction

The liver is the most frequently injured intra-abdominal organ because of its location within the abdomen and its fragile material properties [1]. In 2007, 1.7 million car accidents in the United States resulted in injury (National Highway Traffic Safety Administration) with the liver being one of the most commonly injured abdominal organs from motor vehicle accidents [2,3]. Efforts to determine the optimal safety measures for automobile-related accidents have largely relied on crash dummies, which have significant limitations in recapitulating injury impact to humans [4]. Since the 1970s, there have been no substantial changes in assessing injury. Injury assessment reference values (IARVs) proposed by General Motors for dummies in crash tests were determined via force and acceleration calculations and defined a tolerance level of 5% significant injury risk of various organs [5,6]. An improved, more cost-effective alternative to assess organ damage during car crash situations is the development of computational models that represent the human body and more accurately predict the risk of human tissue/organ injuries. Recent work in developing a geometrically correct “virtual human” has been performed with the goal of measuring bodily trauma in automobile accidents [7,8,9,10]. 

Besides an anatomically relevant mesh, the development of a biofidelic computational model requires knowledge of the mechanical properties of many human tissues and organs under different loading conditions, especially in high-impact situations. Quasi-static biomechanical characterizations of soft tissues have been performed since the 1970s to determine the mechanical properties of various tissue types; however, regarding the response of tissues that may be subjected to high-impact situations such as automobile accidents, sport injuries, and blunt trauma, these quasi-static tests are limited and cannot be extrapolated to high-rate applications. Mechanical testing therefore must be performed at higher strain rates to properly describe the tissue’s response during blunt force impacts [11,12].

A standard protocol has not been well established for high strain rate mechanical testing on liver tissues. Sparks et al. customized a drop tower in which a weight was dropped onto a whole human liver organ, resulting in average strain rates up to 62 s^−1^ [13]. Others have used indentation instruments to generate strain rates up to 200 s^−1^ [14,15]. This study considered the split-Hopkinson pressure bar (SHPB) apparatus in an effort to establish a methodology of high strain rate testing of soft tissues. The SHPB apparatus has the ability to apply compressive stresses at high strain rates (100–10,000 s^−1^) [11] and has been widely applied in testing of metals and inorganic polymers [16,17]. Elastic wave propagation in the SHPB system can be analyzed based on the principle of superposition of waves and the elastic wave propagation theory of classical mechanics. As a result, the stress, strain, and particle velocity can be estimated by analyzing the incident wave and the reflected wave at any cross-section [18].

When the SHPB is used for testing soft tissues, many issues must be considered to generate consistent and accurate data. The mechanical impedance of soft materials is extremely low compared to conventional metallic bars and, therefore, confound proper interpretation of the data. Incorporation of polymeric bars into the SHPB setup has allowed for testing of soft materials such as rubber and biological tissues, of which the acoustical impedance matches more closely with that of the softer polymeric bars. Unlike conventional metallic bars, polymeric bars do not impede wave translation and enable a smooth translation of energy generated by the impact of the incident bar and the soft specimen; this results in smoother, more noise-free curves [12]. A few groups have applied the SHPB apparatus for soft tissue biomechanics experimentation. For example, Song et al. tested porcine muscle along two perpendicular directions at dynamic strain rates up to 3700 s^−1^ using the SHPB apparatus. They found that both directions showed a nonlinear, strain rate-dependent behavior [12]. Similarly, Van Sligtenhorst et al. used a polymeric SHPB apparatus to obtain the mechanical response of bovine muscle at strain rates up to 2300 s^−1^ [11]. As they varied strain rates, they observed strain rate dependency. Recently, Pervin et al. used an aluminum SHPB apparatus to evaluate bovine liver tissue at strain rates ranging from 1000–3000 s^−1^, and they also found the tissue to exhibit a nonlinear, strain rate-dependent response [19]. 

The objective of the present work is to investigate the tissue behavior of porcine liver at high-rate impacts using a custom-made PSHPB coupled with finite element analyses. The protocol for testing soft biological materials using PSHPB has been adopted from previous studies [18,20,21]; however, the protocol for procuring and preparing porcine liver samples was developed in-house. Both experimental results and computational simulations of liver tissue under high strain rate conditions will provide the framework to be incorporated into a human model. This model in the future will be implemented to optimize the automobile safety measures to reduce the risk of human injuries and death in high-impact situations.

## 2. Methodology

### 2.1. Sample Preparation

**Figure 1:**
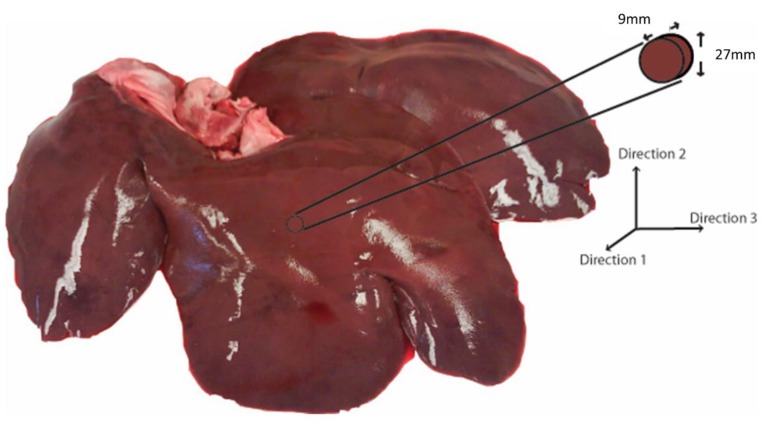
Three orthogonal directions (1, 2, and 3) based on porcine liver anatomy. Representative sample geometry and size.

Porcine livers from healthy adult pigs were obtained from a local abattoir (age range: 6–9 months; weight range: 250–350 lbs.; sex: male). The specimens were stored in phosphate-buffered saline (PBS) (Sigma-Aldrich, St. Louis, MO, USA) at 4 °C soon after extraction and were transported to the laboratory. All testing was performed within 12 h of extraction. For PSHPB application, the tissue sample was carefully extracted to maintain a certain shape and size. A bar diameter larger than the sample size was important to ensure that most of the energy was transmitted through the sample [11]. Testing was performed on samples with aspect ratios ranging from 1:1 to 3:1, and it was determined that an aspect ratio of 3:1 produced consistent data (results not shown). Thus, a cylindrical die of 30 mm inner diameter was used to cut disc-shaped samples to approximately 27 mm in diameter and 9 mm thick for an aspect ratio of 3:1 (Figure 1). The aspect ratio and size corresponded well with previous studies [11,22]. In order to extract samples in different directions, a 9 mm slice was cut in the appropriate direction, laid on its side, and then punched out with the cylindrical die. The axis of the disc-shaped sample was aligned along one of the three orthogonal directions.

### 2.2. High Strain Rate Testing Using a Polymeric Split-Hopkinson Pressure Bar (PSHPB)

**Figure 2:**
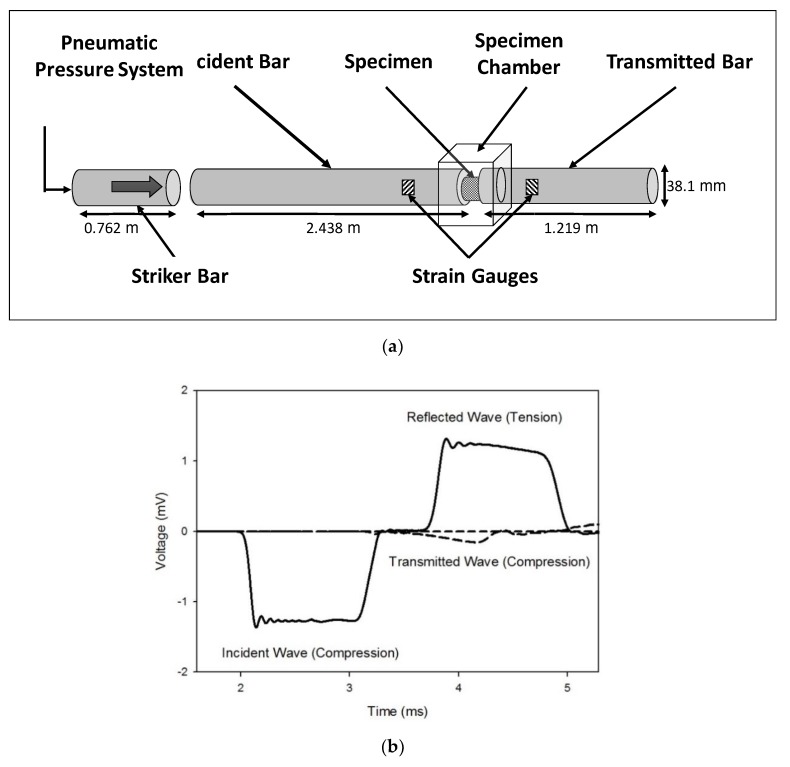
(**a**) Schematic of the polymeric split-Hopkinson pressure bar (PSHPB) apparatus. (**b**) Incident, reflected, and transmitted waves obtained from PSHPB testing on porcine liver tissue.

High strain rate testing was performed as per previously established protocol [23,24,25]. The PSHPB, made of commercially extruded natural polycarbonate (PC 1000) rods, was composed of a striker bar, an incident bar, and a transmitted bar with lengths of 0.762, 2.438, and 1.219 m, respectively, and a diameter of 38.1 mm (Figure 2a). A cylindrical specimen was placed between the incident and transmitted bars, and the striker bar was propelled at a specified velocity by means of a pneumatic pressure system. The sample was compressed in the z-direction. The z-direction was normal to the 27 mm diameter surface of the liver specimen. 

As the striker bar impacted the incident bar, a compressive wave (incident wave) was generated and propagated down the incident bar where it reached the specimen, which caused compression of the specimen. At this point, a portion of the wave was reflected back into the incident bar as a tensile wave (reflected wave). The remainder of the compressive wave (transmitted wave) was transmitted through the specimen and into the transmitted bar (Figure 2b). The incident, reflected, and transmitted waves were measured by two strain gauges—one gauge on both the incident and transmitted bars. The PSHPB experimental setup was based on the following assumptions: (i) the specimen undergoes uniform and uniaxial stress during deformation; (ii) the incident and transmitted bars are elastic; (iii) the edges of the bars in contact with the specimen remain flat and parallel; (iv) the incident, transmitted, and reflected waves undergo minimal dispersion as they travel along the bars; and (v) strains measured at the surface of the bars are indicative of those throughout the cross-section [26]. The theory behind the SHPB setup and the constitutive true stress–strain relationship of the sample deformation is briefly discussed in Appendix A. The experimental setup also included a laser speed meter for monitoring the incident bar speed and DAQ modules for data acquisition. Data were processed via DAVID Viscoelastic Software [18]. 

Cylindrical samples were extracted from three orthogonal directions based on porcine liver anatomy (Figure 1). For evaluating strain rate sensitivity, samples were extracted along Direction 1 (Figure 1), and strain rates of 350 (*n* = 4), 550 (*n* = 4), 1000 (*n* = 4), and 1550 s^−1^ (*n* = 5) were applied. The range of strain rates was chosen based on the deformation rate from impact at 55 km/h (more than 1000 s^−1^) [27]. To evaluate the directional dependence (anisotropy) of tissue behavior, samples were dissected along three orthogonal directions (Directions 1, 2, and 3; *n* = 4 for each direction) and tested at a strain rate of 350 s^−1^. For each test, a sample was glued between the incident and transmitted bars using cyanoacrylate glue (Cemedine, Japan) [28,29]. Liver tissue was kept moist with PBS throughout the testing procedure, and testing was carried out at room temperature (21–23 °C).

Statistical analyses of three parameters, namely the tangent modulus, peak stress to valley stress ratio, and ultimate stress to valley stress ratio, were conducted using the SigmaStat 3.0 software (SPSS, Chicago, IL, USA). A one-way analysis of variance (ANOVA) method was used for statistical analysis on the two parameters, and a Holm–Sidak test was used for post hoc comparisons. A paired Student’s t-test was used to calculate the mechanical difference between the two parameters at different strain rates. For *p* < 0.05, the mechanical difference at various strain rates, for a particular parameter, was considered to be statistically significant.

### 2.3. Microstructural Analysis

To assess the microstructural characteristics of liver tissue along different orthogonal directions, samples were dissected along each orthogonal direction (1, 2, and 3) corresponding to the orientation of samples used for high strain rate testing. Liver samples were fixed in 10% neutral buffered formalin and dehydrated in a graded ETOH series. Samples were then embedded in Paraplast with CitriSolve as a transitional fluid, sectioned to a thickness of 7 μm, and subjected to hematoxylin and eosin (H&E) staining. In H&E staining, liver cell nuclei were stained black/purple, and extracellular matrix proteins were stained pink.

ImageAnalyzer v.2.2-0 software (CAVS, Mississippi State University, Starkville, MS, USA) was used for microstructural analyses of histological images from samples cut along each orthogonal direction [30]. The parameters obtained for each image during analysis included the following: object count, cell nuclear density, area fraction of cell nuclei, mean area of cell nuclei, and mean nearest neighbor distance (nnd). Total cell nuclei area was a measure of the total area of all cell nuclei, and area fraction was the ratio of total cell nuclei area to total image area. Mean area represented the average area of cell nuclei, and object count was the number of nuclei present in the image. Cell nuclear density equaled the object count divided by the total image area. Mean nnd was a measure of the average distance between neighboring nuclei.

### 2.4. Finite Element Modeling

Similar to Prabhu et al. [25], finite element (FE) simulations (ABAQUS/explicit solver version 6.9) of porcine liver high-rate tests were conducted to better understand the behavior of the liver tissue under high-rate compression. The bars in the FE simulations were modeled as an elastic polycarbonate material (Young’s modulus of 2391 MPa and Poisson’s ratio of 0.36) [25]. The hyperelastic and inelastic behaviors of liver tissue were fitted (using partial least square fitting in Matlab^®^) with a phenomenological internal state variable (ISV) material model developed by Bouvard et al. (Mississippi State University TP, version 1.0) [31,32]. The constitutive model (MSU TP version 1.1) used in this study captured both the instantaneous and long-term steady-state processes during deformation and could admit microstructural features within the internal state variables. With the microstructural features, the internal state variable model used for this study can eventually capture and predict history effects in tissues. In the absence of the microstructural features, other constitutive models should be able to show the nonuniformity of the stress state under the high-rate loadings exhibited here since no varying history was induced. Data from high strain rate PSHPB tests were utilized to calibrate the numerical model (referred here as MSU TP 1.1) using MATLAB (MathWorks Inc., Natick, MA, USA, 2010). Curve fitting (MSU TP 1.1) was performed for experimental data obtained at 205 s^−1^, and strain rate dependency of the material model was validated with the stress–strain response of the tissue at 550 s^−1^. Subsequent to the previous step, the model calibration was two-fold. The first step in calibration was performed such that the experimental and FEA strain gage data analyzed through DAVID Elastic (resulting in true stress–strain curves) were in good agreement. In the second step of calibration, the strain gage measurements from the SHPB experiment and FE simulation ere also correlated. Thus, correlation was performed for both strain measurements and the stress–strain of the specimen. It was noteworthy here that the material point simulator, being 1-D, gave a 1-D stress state for calibration, while the stress state in experiments and FE simulations were 3-D. The difference in the loading direction stress σ_33_ between the material point simulator, experiment, and FEA was due to the presence of a 3-D stress state in the experiment and FEA. So, the model calibration was performed through an iterative optimization scheme where model constants were varied appropriately until the experimental and specimen volume averaged FE simulation σ_33_ matched. The values for the material constants for MSU TP 1.1 are found in Table 1. Using calibrated data from PSHPB experiments, several FE simulations at strain rates of 350, 550, 1000, and 1550 s^−1^ were performed (ABAQUS/explicit solver version 6.9 [33]). The finite element model was composed of 22,010 hexahedral elements with the specimen containing 9200 elements. Mesh refinement was conducted to analyze the convergence of computational solutions. Boundary conditions included specified initial velocity for the striker bar. The finite element model simulated the experimental PSHPB setup corresponding to different strain rates, and a “contact” was defined between the surfaces of the specimen and polycarbonate rod.

## 3. Results

**Figure 3:**
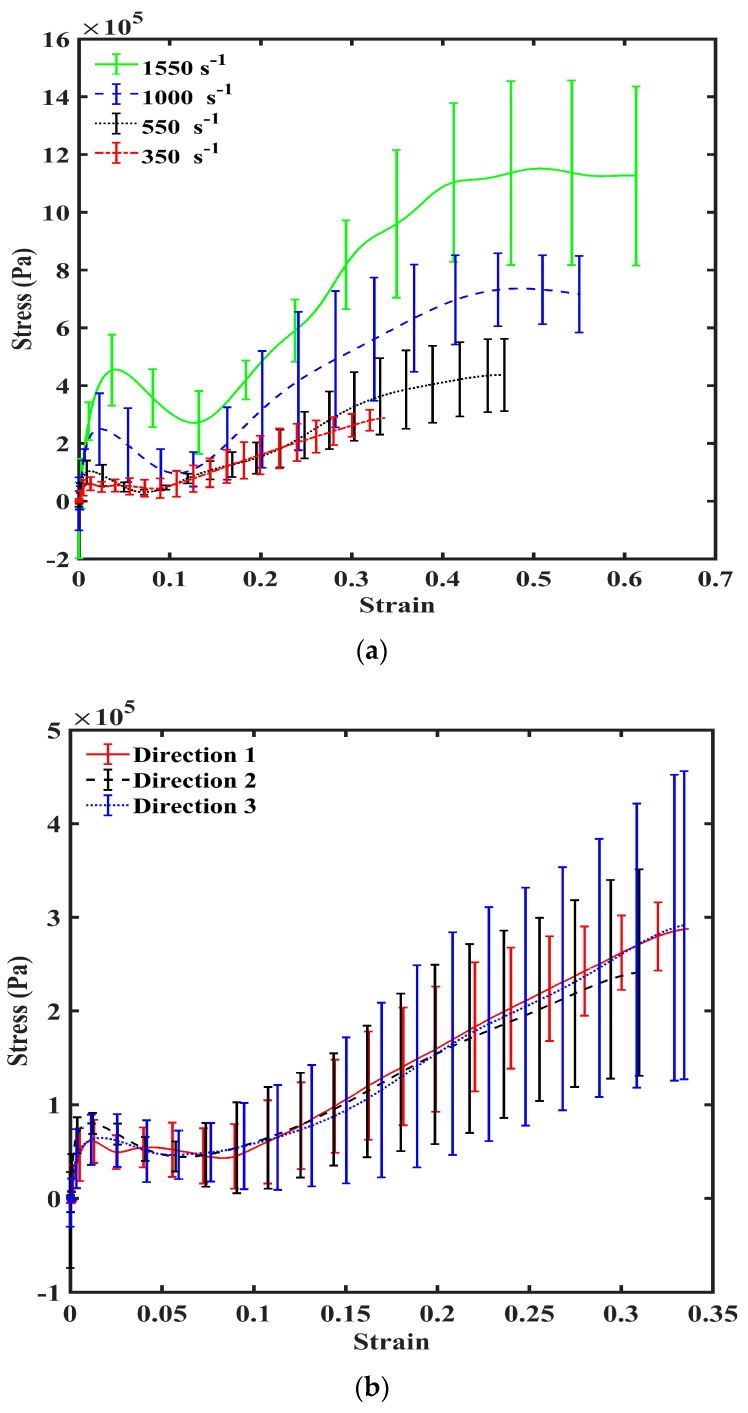
(**a**) True stress–strain response of porcine liver tissue at 350, 550, 1000, and 1550 s^−1^ in Direction 1. *n* = 4 for 350, 550, and 1000 s^−1^; *n* = 5 for 1550 s^−1^. Error bars indicate standard deviation. (**b**) Mechanical true stress–strain response of porcine liver tissue at 350 s^−1^ in Directions 1, 2, and 3 (*n* = 4) illustrating isotropy. Error bars indicate standard deviation.

PSHPB experiments showed that liver tissue had a strain rate-sensitive behavior under high-rate compression (Figure 3a). Stresses were significantly higher as the strain rate increased from 350, 550, 1000, to 1550 s^−1^. The resulting stress–strain behavior showed that the liver tissue exhibited an initial stiffening behavior, which was followed by softening. After softening, tissue hardening took place until yielding and ultimate failure. The nonmonotonic stress–strain behavior described above was apparent for all four strain rates (350, 550, 1000, and 1550 s^−1^).

To further examine the relationship between strain rate and liver tissue’s mechanical response, data analyses of the stress–strain behaviors at 350, 550, 1000, to 1550 s^−1^ were performed by normalizing both the initial peak stress and the ultimate stress to the valley stress (lowest stress value following initial peak). Both the ratio of peak stress/valley stress and the ratio of ultimate stress/valley stress decreased with an increase of the strain rate (Table 2). Increasing strain rate from 550 to 1000 s^−1^ and from 1000 to 1550 s^−1^ yielded significant differences in the peak to valley stress ratios (ANOVA *p* < 0.05). 

**Figure 4:**
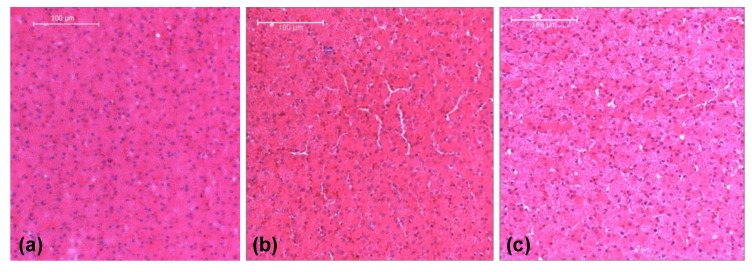
Liver histology revealing the tissue’s homogeneity and isotropy. (**a**) Direction 1, (**b**) Direction 2, and (**c**) Direction 3. Liver tissues were fixed with 10% formalin at the load-free condition.

**Figure 5:**
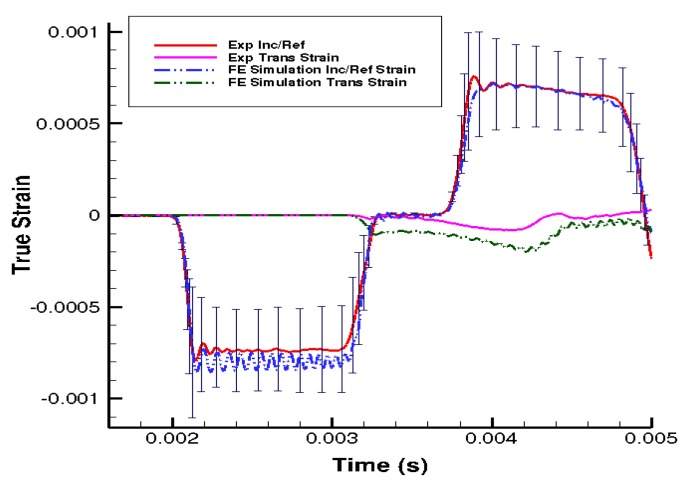
Comparison of the strain in the loading direction from the experimental data and finite element (FE) simulation results at 550 s^−1.^

The stress–strain behaviors of the liver tissues extracted from three orthogonal directions exhibited no significant differences at 350 s^−1^ (Figure 3b). Overall at high strain rates, porcine liver tissue demonstrated nonlinear, inelastic, strain rate-sensitive mechanical responses; all responses were characterized by an initial peak and subsequent hardening until yielding and failure. The isotropic mechanical behavior was verified by H&E staining of the samples, which showed black/purple cell nuclei and pink extracellular matrix of hepatocytes. This study revealed identical ultrastructures along the three orthogonal directions (Figure 4, Table 3). Image analysis of these stained images revealed no differences regarding each of the three directions in terms of cell nuclear density, area fraction of cell nuclei, mean area of cell nuclei, and mean nnd (*p* > 0.05; data not shown here). Figure 5 gives the strain-time responses (along the loading direction) for the experiment and FE simulation. As observed, there was very good correlation between the experiment and the FE model. The comparison of incident, reflected, and transmitted waves between the PSHPB test and the FE simulation showed that the dispersions of the stress waves were assessed appropriately in the FE model. This implied that the elastic assumption for the dispersion of the waves in the polymeric bars of the FE model was appropriate for modelling the high strain rate phenomenon. The high-frequency fluctuations observed in the FE model strain measurements (Figure 5) could be attributed to the “frictionless” nature of the FE model. While the experiment accompanied friction arising from PSHPB clamp contacts that dampened the fluctuation, the FE model neglected such contact from frictional effects as minimal. It can be observed from Figure 5 that the assumption of frictionless PSHB clamp contacts had not compromised the trend and agreement of the FE model strain data with the experimental data. 

**Figure 6:**
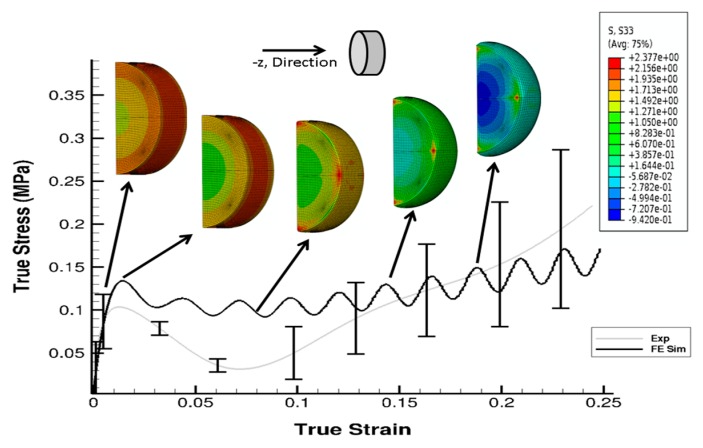
Contour plot (σ_33_) with comparison of σ_33_ from the experiment and the FE simulation at 550 s^−1^. Sample is a solid cylindrical disk, and σ_33_ data from FE simulation were processed in DAVID Viscoelastic Software.

**Figure 7:**
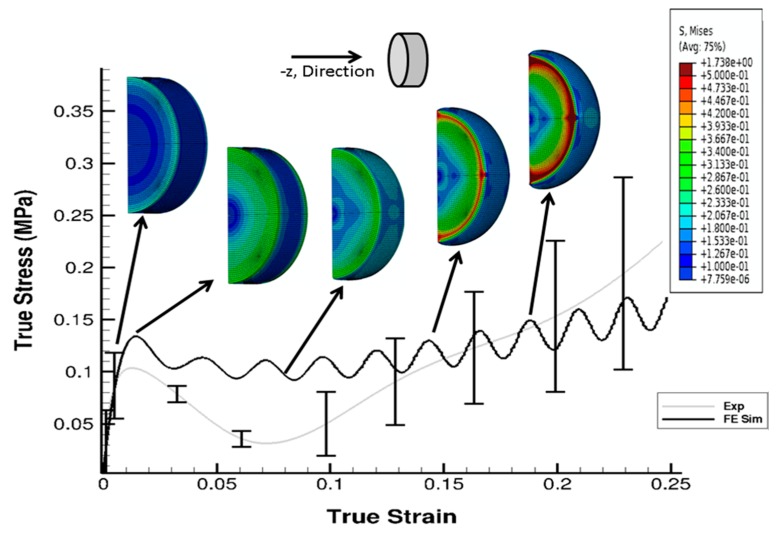
Contour plot (von Mises data) with comparison of σ_33_ from the experiment and FE simulation at 550 s^−1^. Sample is a solid cylindrical disk, and σ_Mises_ data from the FE simulation were processed in DAVID Viscoelastic Software.

**Figure 8:**
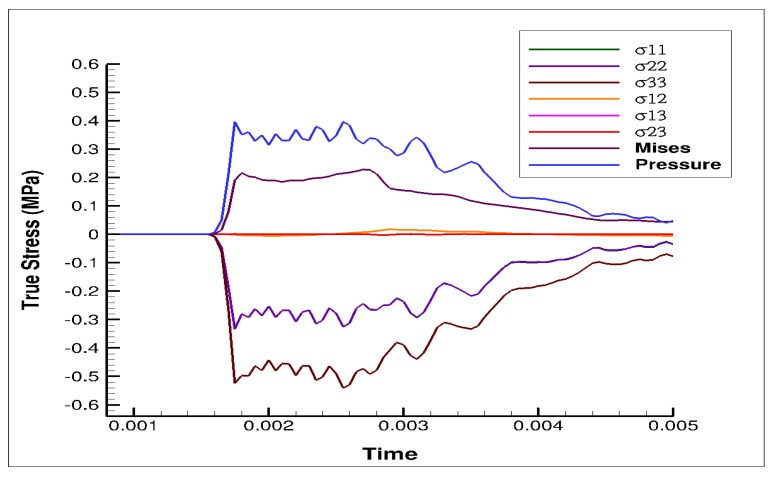
Plot of pressure, von Mises, and stress from different orthogonal directions (σ_11_, σ_22_, σ_33_, σ_12_, σ_13_, and σ_23)_ over time, obtained from the FE simulation at 550 s^−1^. Compressive stresses are negative.

The stress state in the cylindrical liver sample was revealed by the FE model of the PSHPB test at a strain rate of 550 s^−1^ (Figure 6). The loading direction stress (σ_33_) contour plots in the specimen are illustrated in Figure 6. The contour plots of σ_33_ (axial stress) and σ_Mises_ were found to vary dramatically during the initial hardening trend. The ε_33_ and von Mises contours of the sample revealed a nonuniform stress state throughout testing (Figure 6 and Figure 7). The stresses varied over time as shown in Figure 8.

## 4. Discussion

Hopkinson bar testing on soft tissues is a relatively new effort with few reports within the recent decade [11,12,22]. To obtain valid and accurate stress–strain data and further establish Hopkinson bar testing as the conventional high strain rate test for soft tissues, many variables were evaluated in this study. For example, specimen aspect ratio was an important factor for consideration so as to avoid unequal stress distribution in the sample and nonequilibrated input/output forces from an overly wide sample. Van Sligtenhorst et al. suggested an optimal aspect ratio of approximately 2:1 for bovine muscle tissue samples to produce equal stress distributions through the cross-section [11]. For choosing the appropriate aspect ratio, one must take into consideration two trends: (i) increasing the specimen aspect ratio can cause an increase in radial inertial effects; however, (ii) decreasing the specimen aspect ratio below a certain level could lead to nonuniform deformation along the longitudinal axis of the sample [11]. Van Sligtenhorst et al. and Song et al. showed the effects of specimen aspect ratio on the accuracy of PSHPB testing, and as a result, samples for the present study were prepared with the previously stated geometric criteria in the experimental design. By generating consistent, repeatable data that reflected the intrinsic mechanical response of liver tissue, the optimal aspect ratio for liver tissue was determined to be 3:1.

The obtained true stress-true strain curves showed a nonmonotonic characteristic overall (Figure 3a,b), which was similar to recently reported data from drop tower compression testing of human liver tissues at strains rates up to 62 s^−1^ [13]. Results from both studies indicated a loading path that initiated with a sharp stiffening response, followed by softening, subsequent hardening, and then yielding until ultimate failure. It is notable that this initial stiffening does not appear in stress–strain plots obtained in the regime of low strain rates (<10 s^−1^), which often exhibit a monotonic, concave-upwards stress–strain relationship [28,34]. Song et al. hypothesized that the stiffening was purely a result of inertia [12]; however, this may or may not be the complete conclusion. Similarly, Sparks et al. cited inertia as the main factor in initial stiffening but included dynamic changes in specimen geometry during loading as a factor in stiffening [13]. Additionally, our study showed that the initial hardening trend observed in the high strain rate response of soft biological materials may completely be due to inertial effects, but part of it could also be attributed to the intrinsic behavior of the material [23]. Further, Prabhu et al. also asserted that the high water content (70–80%) of soft biological materials contributed to part of the initial hardening observed in high strain rate responses of soft biomaterials [23]. 

As observed in Figure 6 and Figure 7, the high-frequency oscillatory behavior of the FE simulation stress–strain response was higher than that of the experimental response. The experiment, which represented more of a real-world scenario, encountered the lower levels of such high-frequency oscillations. In real life experimental systems, energy dissipation mechanisms act as a damping source, but in an FE model, which is an undamped system simulating a real world damped experimental system, proportional damping is commonly used to account for dissipative mechanisms (Appendix B). 

It is interesting to note that high strain rate testing of different tissues results in different degrees of initial stiffening. The results obtained by Prabhu et al. for brain tissue and by Clemmer et al. for liver tissue at high rate compression demonstrated a higher initial hardening peak when compared to liver data reported in this study [23,24]. The above observation leads to a hypothesis correlating the initial stiffening with concentration of cellular content/water content [23]. Specifically, tissues with higher cellular content have a higher initial stiffening peak than those of a more fibrous nature (e.g., brain > liver > tendon). One of the future aims of this research is to characterize various soft tissues in an effort to confirm that the initial stiffening effect is actually an accurate representation of tissue behavior under high strain rate testing. 

To our knowledge, no studies involving high strain rate Hopkinson pressure bar testing of porcine liver tissue exist. Moderate strain rate testing (20–62 s^−1^) on human liver tissue was performed by Sparks et al. (2008), and they showed similar trends in stress–strain plots, despite the difference in methodology, in which a drop tower technique was used on intact human livers [13]. Though these strain rates were considered as fairly high in the report, they were relatively low compared to the strain rates obtained in the present study. Through repetition of testing using the PSHPB apparatus, various input velocities of the striker bar resulted in consistent strain rates in the porcine liver tissue (Table 4). Using the PSHPB method, striker bar speeds of approximately 6.5–17 mph corresponded to strain rates of 350–1550 s^−1^ in the liver tissue. For accurate replication of car crash scenarios, speed is a critical factor, and the impact speeds employed in the present study are more representative of speeds at which blunt trauma situations, such as those resulting from automobile accidents, occur.

The anisotropic mechanical response of liver tissue was also addressed in this study. The isotropy (or anisotropy) of liver tissue at high strain rates has not been well accepted in the present literature; therefore, evaluating this material property for modelling purposes was necessary. Previous work on high strain rate testing of bovine liver along two perpendicular directions determined that bovine liver behaved isotropically [19]. The present study extends this work to include three orthogonal directions in porcine liver tissue, along which high strain rate tests and histological analysis were performed. High strain rate mechanical testing clearly showed that no difference existed among stress–strain behaviors from testing along three orthogonal directions. Histological analysis of liver tissue, which examined characteristics as cell nuclear area, cell count, and mean distance between neighboring cells, revealed microstructural similarities among samples oriented along Directions 1, 2, and 3. These findings suggest the liver is an isotropic medium. Although exact mechanisms for the isotropic response are not yet clear, future studies into the contributions of the extracellular matrix may be insightful. The heterogeneous response of the liver tissue behavior during plastic deformation in an experimental setup is specimen-, microstructure-, or location-dependent, which is difficult to integrate in a simulated homogenous finite element model. In the PSHPB experimental setting, porcine liver specimens were glued to the setup, whereas the finite element model incorporated only minimal contact between the specimen and the polycarbonate rod. Even though utilization of glue during biomechanical testing of porcine liver tissue is not uncommon [34], it is difficult to quantitatively delineate the mechanical role of glue in PSHPB; hence, it was assumed to be constrained-body contact that was applied in the FE studies. Furthermore, high water content is essential for viscoelastic responses of soft tissues [1]. Incorporation of this mixture theory-based viscoelastic response in the material model can potentially capture the “softening” and further “hardening” responses; however, this component is beyond the scope of the current 1D material point simulator and will be addressed in future studies.

## 5. Conclusions

The use of a PSHPB apparatus for high strain rate testing of porcine liver tissue reveals the inelasticity, isotropy, and strain rate sensitivity of liver tissue. In conclusion, (i) the liver tissue response at high-rate compression was characterized by an initial hardening peak, followed by softening, and then by strain hardening to failure; (ii) the liver mechanical stress–strain behavior increased as the applied strain rate increased; and (iii) isotropic high-rate material behavior was observed along all three orthogonal directions and was confirmed by the liver histological microstructure.

In addition to these three conclusions, some other important points are worth mentioning. The wave propagation predicted by the finite element PSHPB simulation was consistent with the experimental results, thus substantiating the present results of the PSHPB. However, the computational simulation of the PSHPB process also showed that a uniform stress state was not fully achieved in the cylindrical sample. This limitation implies that future work is warranted to perfect the PSHPB technique in soft tissue high-rate characterization.

High strain rate tests conducted using an SHPB apparatus show that the porcine liver is strain rate-dependent (Figure 3a). The anisotropy of the material at a high rate is marginal but marked with high variation in the sample-to-sample mechanical behavior (Figure 3b). The material response is marked by an initial hardening effect, followed by a softening trend, and then further hardening at larger strains (Figure 3a,b). Simulations of the PSHPB test in ABAQUS/explicit solver (version 6.9) showed that the axial stress σ_33_ was primarily concentrated in the central region of the specimen (Figure 6 and Figure 7). Specimen volume-averaged FE simulation results indicated that a homogeneous stress state was not maintained during specimen deformation (Figure 6 and Figure 7). The range of stresses exhibited by the specimen in the FEA can be observed in Figure 8.

This novel approach using polymeric bars for high-rate impact of porcine liver tissue serves as a benchmark for future high strain rate testing of soft tissues. Experimental data coupled with the finite element model can be implemented in large-scale, computational models of the human body for simulation of high strain rate scenarios, such as automobile accidents, for validating the efficacy of various safety features.

## Figures and Tables

**Table 1:** Values of material constants for liver material using the MSU TP 1.1 model.

Model/Material Constants.	Values
μ (MPa)	29
K (MPa)	12,492
γ_vo_ (s^−1^)	99,209.5
m	1.1
Y_o_ (MPa)	2
α_p_	0
λ_L_	7
μ_R_	0.168627
R_s1_	1.4
h_o_	31
ζ^o^_1_	0
ζ*_sat_	0.01
ζ*_o_	0.3
g_o_	0.07
C_κ1_ (MPa)	0.4
h_1_	0
e^o^_s2_	0
e^sat^_s2_	0.4
C_κ2_ (MPa)	0

**Table 2:** Ratio of peak stress/valley stress and ratio of ultimate stress/valley stress shows an overall decreasing trend along with the increase of the strain rate. (*n* = 4).

Strain Rate (s^−1^)	Mean Peak Stress/Valley Stress	Mean Ultimate Stress/Valley Stress
350	5.37 ± 4.59	21.92 ± 16.39
550	3.55 ± 1.56	13.36 ± 6.09
1000	3.00 ± 0.84	12.09 ± 3.56
1550	1.42 ± 0.24	12.48 ± 2.12

**Table 3:** Image analysis results from Figure 4a–c revealing the tissue’s homogeneity and isotropy.

	Direction 1	Direction 2	Direction 3
**Objects**	797	795	803
**Cell nuclear density (/mm^2^)**	4.08 × 10^3^	5.91 × 10^3^	4.11 × 10^3^
**Area fraction of cell nuclei**	9.79%	13.4%	8.06%
**Mean area of cell nuclei (μm^2^)**	23.98 ± 15.58	22.6 ± 15.71	19.59 ± 7.73
**Mean nearest neighbor distance (nnd) (μm)**	9.62 ± 3.14	8.44 ± 2.40	9.29 ± 3.12

**Table 4:** Correlation between striker bar speed and resultant strain rate of porcine liver tissue in high-rate tests.

Velocity of Striker Bar (mph)	Strain Rate of Liver Tissue (s^−1^)
6.487	350
9.843	550
13.645	1000
17.001	1550

## Disclaimer

This report was prepared as an account of work sponsored by an agency of the United States Government. Neither the United States Government nor any agency thereof, nor any of their employees, makes any warranty, express or implied, or assumes any legal liability or responsibility for the accuracy, completeness, or usefulness of any information, apparatus, product, or process disclosed, or represents that its use would not infringe privately owned rights. Reference herein to any specific commercial product, process, or service by trade name, trademark, manufacturer, or otherwise does not necessarily constitute or imply its endorsement, recommendation, or favoring by the United States Government or any agency thereof. The views and opinions of authors expressed herein do not necessarily state or reflect those of the United States Government or any agency thereof.

## Appendix A. Working Principle of Split Hopkinson Pressure Bar (PSHPB)

The theory for PSHPB is based on classical mechanics of elastic wave propagation in the bars and on the principle of superposition of waves. In elastic wave propagation theory, stress, strain, and particle velocity caused from a single pressure wave (here compressive) are proportional to each other. Hence, knowledge of a single pressure wave at any cross-section of the bars enables us to calculate the wave nature at any other cross-section. Using the knowledge of incident wave and reflected wave at any cross-section (and through the principle of superposition), stress, strain, and particle velocity can be calculated. Here the stress, the strain, and the particle velocity are simply the sum of those related to the incident wave and reflected wave, which are in opposite directions [18]. The specific wave energy ($W_{s}$) absorbed by the specimen is given by

(A1) $$W_{s}=W_{i}-(W_{o}+W_{r}).$$

This is the equation for energy balance. Here *W_i_* is the energy of the incident wave, *W_o_* is the energy of the transmitted wave, and *W_r_* is the energy of the reflected wave. Stress state homogeneity and balance of input and output are assumed. The bar’s response, being elastic in nature, can be used to calculate the specimen’s inelastic response through the energy balance of the stress waves. The expression for true stress–strain for a PSHPB experiment is given as:

(A2) $$\sigma_{t}(t)=\frac{F(t)}{S_{s}(0)}(1-2\nu \varepsilon_{n}(t)).$$

With true strain defined as

(A3) $$\varepsilon_{t}\left(t\right)=-ln\left(\frac{1}{1-\varepsilon_{n}\left(t\right)}\right),$$

with

(A4) $$\varepsilon_{n}(t)=\int_{0}^{t}{\dot{\varepsilon }}_{n}(t)dt,$$

(A5) $${\dot{\varepsilon }}_{n}(t)=\left(\frac{V_{i}(t)-V_{o}(t)}{l_{s}(0)}\right),$$

where *ε_t_*(*t*) is the true strain (A2), *ε_n_*(*t*) is the nominal strain (A3), ${\dot{\varepsilon }}_{n}(t)$ is the nominal strain rate (A4), *l_s_*(0) is the initial length of the sample (undeformed), *F*(*t*) is the force, *V* is the velocity, *S_s_*(0) is the initial cross-sectional area of the sample, and *ν* is Poisson’s ratio (which is equal to 0.5 for incompressible materials). Subscript *i* and *o* represent variables on the incident and transmitted bars, respectively. The above formulae are applicable to compression with positive strains.

## Appendix B. Mechanism of Rayleigh Viscous Damping

Real world dissipation mechanisms, such as friction, vibration, etc., in a high strain rate experiment are hard to quantify and are simulated using an FE model. However, a generalized damping mechanism, called Rayleigh or classical damping, can be introduced to simulate frictional and vibrational dissipations. Rayleigh damping was introduced as a specific type of viscous damping, which was calculated as a linear combination of mass matrix (*M*) and stiffness matrix (*K*). The equation for viscous damping matrix is as follows:

(A6) $$C=\alpha_{R}M+\beta_{R}K,$$

where *α*_R_ and *β*_R_ are the coefficients of *M* and *K*, respectively, and are real scalars (A6).

The observed difference in the high-frequency oscillatory waves of the FE simulation in comparison to the experimental data (Figure 5) arose from the fact that frictional and vibrational dissipations were simulated in the FE model through viscous damping. Viscous damping is a numerical damping mechanism to account for experimental dissipations.
